# Improving Teamwork Competencies in Human-Machine Teams: Perspectives From Team Science

**DOI:** 10.3389/fpsyg.2021.590290

**Published:** 2021-05-24

**Authors:** Kimberly Stowers, Lisa L. Brady, Christopher MacLellan, Ryan Wohleber, Eduardo Salas

**Affiliations:** ^1^College of Business, The University of Alabama, Tuscaloosa, AL, United States; ^2^College of Computing and Informatics, Drexel University, Philadelphia, PA, United States; ^3^Soar Technology, Inc., Ann Arbor, MI, United States; ^4^School of Social Sciences, Rice University, Houston, TX, United States

**Keywords:** human-machine team, artificial intelligence, third wave AI, explainable AI, teamwork

## Abstract

In response to calls for research to improve human-machine teaming (HMT), we present a “perspective” paper that explores techniques from computer science that can enhance machine agents for human-machine teams. As part of this paper, we (1) summarize the state of the science on critical team competencies identified for effective HMT, (2) discuss technological gaps preventing machines from fully realizing these competencies, and (3) identify ways that emerging artificial intelligence (AI) capabilities may address these gaps and enhance performance in HMT. We extend beyond extant literature by incorporating recent technologies and techniques and describing their potential for contributing to the advancement of HMT.

## Introduction

Human-machine teaming (HMT)^1^ is increasingly relevant to a variety of modern industries, domains, and work environments. Nearly a decade ago, Amazon added robots to their warehouse facilities to participate in stocking (The Future of Work, 2019). More recently, Google initiated a research program to improve human-machine collaboration (Knight, 2017). These examples and others (e.g., IBM, Facebook; see Davenport, 2018) have demonstrated that machine agents, or machines capable of perceiving and acting upon the world autonomously (Russell and Norvig, 2009), can improve human and organizational performance by providing opportunities for increased safety and productivity.

Effective HMT is contingent upon the success of complex interactions between human and machine agents, and between these agents and their environment (Stowers et al., 2017). However, it is difficult to create machine agents that have the advanced competencies (i.e., knowledge, skills, and abilities) necessary to support these complex interactions (Sukthankar, et al., 2012). Consequently, not all HMT results in heightened performance at the individual, team, or organizational level. In their respective literatures, teams researchers (e.g., Salas et al., 2009) and computer scientists (e.g., Klein et al., 2004; Ososky et al., 2012; Seeber et al., 2020) have identified competencies that are important for successful teaming, but efforts to identify promising new technologies in this area have been limited. Thus, there is a need to explore recent technological developments that may contribute to the advancement of HMT research and practice regarding effective human-machine collaboration.

In this perspective, we (1) briefly summarize the state of the science on critical team competencies identified for effective HMT, (2) highlight gaps preventing machines from fully realizing these competencies, and (3) identify emerging artificial intelligence (AI) capabilities that show promise for enhancing these competencies in machine agent teammates. Our goal is to show how HMT can integrate cutting edge advancements from computer science to improve capabilities of machines to function as teammates.

## The Evolution of Human-Machine Teams

Psychologists and engineers have long explored the use of machines to augment and improve human task performance (Fitts, 1951; Dekker and Woods, 2002). In early work, machines operated as tools to facilitate taskwork by automating physical (e.g., product assembly) and cognitive (e.g., text generation) tasks. The goal of the machine was to improve the overall HMT performance (Dekker and Woods, 2002). Historically, the sociotechnical systems approach (Trist and Bamforth, 1951; Cherns, 1976) guided work design for HMT (Trist, 1981). According to this perspective, the human represents the social subsystem, and completes tasks using resources within the technical subsystem, which is represented by the machine (Eason, 2009).

As machines gained intelligence and the ability to adapt in their interactions with humans, researchers (e.g., Parasuraman and Riley, 1997) developed guidelines regarding the appropriate design and use of machines, including guidelines for their autonomy and adaptivity (e.g., Parasuraman et al., 2000). Broader frameworks describe the human, machine, and contextual inputs, and the resulting processes and states that define human-machine performance (Pina et al., 2008; Stowers et al., 2017). These frameworks also highlight the temporal nature of interaction between humans and machines.

In the last decade, the conversation has shifted from machines as tools to machines as teammates (Phillips et al., 2011; Seeber et al., 2020). The introduction of machines as a component of the social – rather than solely the technical – system has resulted in new design-related challenges (Sukthankar et al., 2012). For example, once machines attain a certain level of intelligence, humans tend to judge machines in much the same way they do their fellow humans, seeking human likeness where it may not exist (Nass et al., 1995; Groom and Nass, 2007). From the HMT perspective, this has been referred to as “teammate-likeness,” where the human perceives the machine as possessing agency, altruism, task-interdependence, relationship building, sophisticated communication, and shared mental models (SMM; Wynne and Lyons, 2018). Teammate-likeness is contingent on factors such as trust and ability (Schaefer et al., 2016) and may also be contingent on machine cues that imply emotional intelligence (e.g., empathy, perspective taking; Salovey and Mayer, 1990). Given that machines still lack the capacity for true emotional intelligence and other socio-emotional competencies that are on par with humans (e.g., Picard et al., 2001; Erol et al., 2020), creating technologies and techniques that allow machines to live up to this perceived teammate-likeness presents a unique challenge.

## Gaps in Machine Competencies for HMT

It may not be necessary for machines to possess all human socio-emotional competencies to be effective teammates. However, the creation of certain capabilities allows machines to develop attitude-based competencies that have been identified as critical for the optimization of teams (c.f. Salas et al., 2009), such as cohesion and mutual (rather than one-way) trust (Groom and Nass, 2007). Recent work in team science has emphasized three team competencies that are transportable across contexts (Salas et al., 2018), namely communication, coordination, and adaptability (hereafter referred to as adaptation). These competencies, referred to as *transportable teamwork competencies*, are applicable in any effective team, regardless of the team or task environment (Salas et al., 2018).

Although team researchers discuss communication, coordination, and adaptation strictly in the realm of human teams (Salas et al., 2018), others have highlighted their importance to HMT (Stowers et al., 2017; Seeber et al., 2020). These competencies are considered universally relevant collaborative processes (Salas et al., 2018; Seeber et al., 2020). Due to wide applicability across team and tasks types, we feature them in this perspective piece as critical areas, where additional technological advancements could improve HMT performance on a large scale. Here, we describe the state of the science on these competencies in HMT and identify gaps where new technologies can provide benefit.

### Communication

Communication, which refers the process of exchanging information between teammates (Salas et al., 2009), is important for team performance as it contributes to the development and maintenance of SMMs and the successful execution of many necessary team processes, including planning and mission analysis (Salas et al., 2009). In HMT research, the process of information exchange between humans and machines has been examined *via* the concept of transparency, defined as “the quality of an interface (e.g., visual, linguistic) pertaining to its abilities to afford an operator’s comprehension about an intelligent agent’s intent, performance, future plans, and reasoning process” (Chen et al., 2014, p. 2).

Although perfect transparency has yet to be realized in HMT (Nam and Lyons, 2020), machines have gained the capacity to share information with humans and coordinate more effectively in joint tasks (Lyons, 2013; Chen et al., 2014, 2018). This includes using turn-taking (Chao and Thomaz, 2016), which is integral to human-machine fluency (Hoffman, 2019). Features such as turn-taking and the ability to recognize human language (Tellex et al., 2020) can enhance the bidirectionality of communication between humans and machines, making HMT in general more teamlike and efficient (Chen et al., 2018).

Researchers studying effective HMT have identified that the *trust* a human member has in a machine is a critical factor in successful team communication (Nam and Lyons, 2020) as well as HMT fluency (Hoffman, 2019). To that end, researchers have looked at how communication can promote trust, and established guidelines for the effective design of information for promoting trust and overall performance (Chen et al., 2014; Sanneman and Shah, 2020). In addition to guidelines regarding the quantity and design of information, researchers have investigated the quality of information shared by HMT members and have applied frameworks of human situation awareness to understand and improve communication processes, such as the mitigation of confusion (Chen et al., 2014; Stowers et al., 2020).

Despite improvements in communication-related abilities, not all machines apply these skills with equal success, leaving gaps between high-performing machines and high-performing HMTs. For example, machines utilizing neural networks tend to have higher performance than other machines, but generally utilize poorer communication due to their use of distributed statistical representations (Nam and Lyons, 2020). Teaming done with machines utilizing neural networks would benefit from an improvement in explainability (Gunning and Aha, 2019). Technological advancements in the area of explainable AI (XAI) show promise for enhancing transparency between humans and machines that utilize neural networks. In short, XAI is AI that can be understood by humans (Gunning and Aha, 2019; Sanneman and Shah, 2020). A goal of XAI research (Gunning and Aha, 2019) is to identify approaches for communicating AI models and their inferences in a format that human operators can comprehend. This research exposes recent XAI-afforded competencies (e.g., transparency), which contributes to understanding and trust between human(s) and machine(s) (e.g., Nam and Lyons, 2020), thus impacting HMT communication.

Communication in HMT must be a two-way street. Machines must be able to effectively share information in a way that humans can understand, but to do so means that machines must possess the ability to accurately model human comprehension of information. This ties in closely with the second transportable teamwork competency: coordination.

### Coordination

While communication refers to the information-sharing process, coordination in human teams refers to the organization of team members’ knowledge, skills, and behaviors to meet a specific goal (Salas et al., 2009). In HMT literature, coordination is defined as the process through which humans and machines manage “dependencies between activities” (Malone and Crowston, 1990). In effective team coordination, task-relevant information is communicated in a timely manner, while unnecessary communication is avoided. In this way, effective communication processes can be seen as necessary but not sufficient for effective HMT coordination.

Human-machine teaming scholars have identified three requirements for effective coordination (Klein et al., 2005): members must (1) each be reliable and able to predict each other’s behaviors, (2) possess common historical and present knowledge (Clark, 1996), and (3) be able to re-direct or help each other in tasks (Christoffersen and Woods, 2002). To this end, a machine is considered an effective coordinator if it is reliable, directable, able to communicate intentions, and able to recognize status and intentions of other team members (Klein et al., 2005). These qualities allow the machine to be able to engage in the communication and creation of SMM needed for successful coordination (Matthews et al., 2021).

The primary gap in the development of coordination in HMT lies in the degree to which machines can engage in implicit coordination. Implicit coordination, which refers to the process of synchronizing team member actions based on assumptions of what each teammate is most likely to do (Wittenbaum and Stasser, 1996), is helpful in high workload situations as it reduces “communication overhead” (MacMillan et al., 2004), and allows teammates to focus on the task at hand with minimal distraction. While machines currently possess the ability to detect certain implicit cues; e.g., *via* the recognition of facial expressions (Picard et al., 2001), they are limited in their ability to detect contextual cues. For example, because coordination involves a complex and varying presentation of implicit communication cues (Lackey et al., 2011), it is difficult and expensive to support machine cue perception on a human level. Detection, interpretation, and reasoning about these cues from a human perspective (Baker et al., 2011) is imperative to ensure effective coordination.

To this end, machines would benefit from developing a theory of mind (Baker et al., 2011). This would translate observations of teammates’ behaviors into a computational model of what they know/do not know, what their goals and preferences are, what capabilities they have, and what behaviors they might take next. Such a model could then be employed to simulate what a teammate might do in different situations or what options they would prefer their machine teammate to take. This kind of capability would support implicit coordination with teammates by enabling the machine to anticipate its teammates’ behaviors and expectations and then to adapt its own behavior to align with those. We elaborate more on adaptation next, including the state of the science and related opportunities for improvement.

### Adaptation

Adaptation in HMT has been examined in two ways: (1) adaptability (i.e., human-controlled adaptation; Miller et al., 2005) and (2) adaptiveness (i.e., machine-controlled adaptation). For example, adaptability can be achieved by supporting human choice regarding the machine’s role, behavioral parameters, and level of autonomy. The human might decide a machine teammate’s tasking order, such as choosing the next lesson given by an intelligent tutoring system (Chou et al., 2015). However, research has shown that humans do not always effectively allocate tasking to automated systems (Lin et al., 2019). To overcome this human limitation, machines can engage adaptiveness by prompting a human to take control of a task (Kaber et al., 2005), or by assuming control in cases of suboptimal task management by human teammates. However, the latter solution poses a threat to human agency (Wohleber et al., 2020) and must be carefully executed.

In team science, adaptation or adaptability as a teamwork competency is examined more broadly and refers to the adjustment of strategies and behaviors in response to changes in the team’s circumstances (Driskell et al., 2018). In considering adaptation through this lens, machines are capable of detecting changes in the internal team and external environments (Lackey et al., 2011), allowing them to engage both adaptive and adaptable mechanisms as designed. They may also detect some underlying causes of changing environments through common sense reasoning (Morgenstern et al., 2016), though this capability remains limited by the datasets used to train common sense (Hao, 2020).

The limitation of machines having to rely on datasets to train common sense has led researchers to explore “third wave AI” capabilities for informing machine knowledge and adaptation. The Defense Advanced Research Projects Agency (DARPA) has argued that to move beyond knowledge-based AI methods (first wave) and statistical machine learning AI methods (second wave), we need approaches that can integrate both first and second wave methods to support contextual understanding and adaptation (third wave, Launchbury, 2017). Most machine learning systems operate by identifying correlational relationships between variables. In contrast, as part of third wave AI, causal and counterfactual models (Pearl, 2019) aim to understand the causal relationships between variables. By modeling causal relationships, machines can better support counterfactual inference; i.e., they can generalize from observed operating conditions to unobserved conditions.

In the last section, we suggested that developing theory of mind in machines (Baker et al., 2011) would be beneficial to HMT coordination and adaptation. For the adaptation piece to be fully realized, it is necessary for machines to be able to not only recognize their teammates’ knowledge and behaviors, but also anticipate and respond to new knowledge and behaviors should they arise. Machines that create and apply causal links to new scenarios should be able to do this and therefore be more effective at modifying their behaviors to engage in a truly adaptive team.

## Conclusion and a Path Forward

Communication, coordination, and adaptation have been identified as critical to the success of both human teaming and HMT, but machines have yet to fully realize the uniquely human cognitive abilities that are necessary for effective teaming (Matthews et al., 2021). However, there are new AI capabilities that could allow machines to maximize these competencies. These capabilities offer a means for machines to begin meeting the requirements needed for effective collaboration with humans.

The capabilities afforded by recent technological advancements show promise for allowing machines to possess the transportable teamwork competencies identified as universally critical to teams (Salas et al., 2018; Seeber et al., 2020). For example, machines might leverage a theory of mind reasoning capability to build a computational model of their teammate based on observations of their behavior. This model might be used to infer what teammates know, what information they have available to make decisions, and what they are likely to do next – enabling better implicit coordination with humans. This theory of mind model, in conjunction with the ability to generate human-explainable outputs (*via* XAI), will also enable machines to determine when and how best to communicate with teammates, further enhancing the trust and ability that affords machine teammate-likeness. Finally, the creation of causal and counterfactual inference capabilities unique to third wave AI will allow machines to be truly adaptive teammates that possess the ability to recognize and reason about the underlying factors that produce changes in the HMT and environment.

While these new technological approaches show promise, more work is needed to refine them to the level required for effective teamwork. Current AI research focuses on developing specific learning and performance capabilities and often does not incorporate findings or insights from the teaming and HMT literature. For example, consider OpenAI Five, a team of five trained neural-network models that can coordinate together to beat a team of five top human champions at Dota 2, a multiplayer online battle arena game (Berner et al., 2019). While the five machines were able to coordinate effectively with each other, a subsequent match with a HMT showed the machines performed worse when partnered with humans. In examining this phenomenon, Carroll et al. (2019) found that the machines were limited by the knowledge they gained from initial training with fellow machines.

Contrastingly, if the OpenAI Five possessed the capabilities outlined in this perspective piece (XAI, theory of mind, and third wave counterfactual prediction), then they should better support communication, coordination, and adaptation with humans. As this example shows, more work is needed to better understand how insights from human teaming and HMT might be integrated into the development of these emerging machines. Increasing collaboration between computer scientists and HMT researchers in examining these insights would be beneficial. With machines operating at new levels of sophistication, true HMT may become possible at a larger scale than seen before.

## Author Contributions

KS, LB, CM, RW, and ES contributed to the writing and content of this paper. All authors contributed to the article and approved the submitted version.

### Conflict of Interest

RW was employed by company Soar Technology, Inc.

The remaining authors declare that the research was conducted in the absence of any commercial or financial relationships that could be construed as a potential conflict of interest.

## Footnotes

^1^We refer to human-machine teams (HMT) as humans and machines working together to accomplisha goal, with machines being autonomous enough to engage in decision making (Seeber et al., 2020). Limited forms of HMT have already begun, but additional sophistication still needs to be achievedbefore machines can be considered true teammates (Sukthankar et al., 2012).
